# Influence of Environmental Factors on Social Participation Post-Stroke

**DOI:** 10.1155/2019/2606039

**Published:** 2019-01-16

**Authors:** Erin L. Foley, Marjorie L. Nicholas, Carolyn M. Baum, Lisa Tabor Connor

**Affiliations:** ^1^Department of Occupational Therapy, MGH Institute of Health Professions, Boston MA, USA; ^2^Department of Communication Sciences and Disorders, MGH Institute of Health Professions, Boston MA, USA; ^3^Program in Occupational Therapy and Departments of Neurology & Social Work, Washington University in St. Louis, St. Louis MO, USA

## Abstract

**Objectives:**

For rehabilitation professionals to adequately address meaningful participation in social activities with their patients after a stroke, there must be a better understanding of neurobehavior, that is, how neurological impairment and its sequelae and environmental factors support or limit social participation. The current study examines how stroke severity (NIH Stroke Scale), its impact on perceived mobility (Stroke Impact Scale mobility domain), and the environment (MOS Social Support–Positive Social Interactions scale and Measure of Stroke Environment receptivity and built environment domains) influence social participation (Activity Card Sort: ACS).

**Methods:**

A correlational, cross-sectional design examined the relationships among neurological impairment, perceived limitations in activity, environmental factors, and social participation. Participants included 48 individuals who were at least 6 months post-stroke both with aphasia (*N* = 22) and without aphasia (*N* = 26) living in the community for whom all measures were available for analysis.

**Results:**

No differences in social participation were found between those with and without aphasia, though both groups reported a large (25-30%) decline in participating in their prestroke social activities. For the ACS Social Domain activities and ACS Partner to Do With activities (percent retained), 37% and 35% of the variance, respectively, was accounted for by the predictor variables, with only MOS Social Support making an independent contribution to social participation. In this sample, neurological impairment was not a significant correlate of social participation. Additionally, perceived mobility and the built environment were not found to *independently* predict participation in social activities.

**Conclusions:**

Perceived social support was found to predict social participation in individuals living in the community 6 months or greater post-stroke. Focusing on social support during post-stroke rehabilitation may provide an avenue for increased social participation and more successful community reintegration.

## 1. Introduction

In the United States, someone has a stroke every 40 seconds, which equates to approximately 800,000 people per year [1]. Unfortunately, stroke is the leading preventable cause of disability with significant societal cost [1]. Due to the prevalence of stroke and the concomitant societal cost, much research has been done on the prevention and rehabilitation of this condition. Less is known about the extent to which individuals who have experienced a stroke successfully reintegrate into the community and the factors that have an impact on the ability to participate in everyday activities after return home, particularly for people with mild stroke.

Participation is a broad and complex concept that can have different meanings to different people. One of the difficulties in defining participation is that there is no gold standard for ideal participation or for which activities or frequency of participation constitute “full participation.” Full participation is individually defined and relies upon self-report. Therefore, for many individuals with disabilities, meaningful engagement means that they have access to a full range of opportunities for participation unrestricted by their physical, cognitive, or mental health challenges or physical, social, or political environments [2].

The concept of participation specifically for individuals post-stroke has been addressed in numerous studies and literature reviews (e.g., [3–6]). According to Woodman et al.[7], participation is an important outcome to consider for stroke survivors because these individuals often measure their recovery by their ability to participate in the activities that brought meaning to their lives before their stroke. Findings from previous studies indicate, however, that individuals often self-report that they are not successfully participating after a stroke [4], even if their neurological impairment is mild, and feel unprepared and distressed upon discharge from rehabilitation because they cannot participate in life in ways they once found meaningful [3]. Additionally, Mayo et al. [5] found that 72% of individuals who have had a stroke lacked important activity to fill their day and were at risk for social isolation. It is clear then that participation is affected after a stroke; yet, there is still a general lack of understanding regarding the factors affecting participation for these individuals.

Social participation, that is, engagement in activities with family, friends, peers, and community [8] is particularly important to stroke survivors [4, 7, 9, 10]. Woodman et al. [7] described social participation as one of the most unique and important aspects of participation because it is the hallmark of what society perceives as functioning. Additionally, this aspect of participation is particularly important to consider because it has been found to increase health-related quality of life for individuals post-stroke, affecting physical, mental, social, and role functioning, as well as a person's own perception of health and well-being [9]. Commonly, however, individuals who have had a stroke experience a loss of social opportunities, changes in their relationships, and social isolation [7].

Although a variety of stroke-related impairments may affect a person's social participation, aphasia may have a particularly significant impact [10]. Aphasia is a communication disorder that affects approximately 25-40% of stroke survivors [11] and typically results in difficulties with speaking, listening, reading, and writing [12]. Therefore, the communication barrier created by aphasia may uniquely contribute to the ability of an individual to participate in activities with others as suggested by previous studies that focused on people with aphasia (e.g., [10]). Overall, the findings from the existing body of literature demonstrate that stroke can have a complex effect on social participation and that it is essential for rehabilitation professionals to help stroke survivors engage in social activities that they personally find most meaningful [4, 7, 9]. For rehabilitation professionals to achieve this, more research focused on the specific factors that influence social participation is necessary, including those with aphasia.

Previous studies revealed that there is a relationship between participation and environmental factors at all levels, including features of the natural and built world, as well as attitudes of others and social policies that affect people with disabilities [2, 13–17]. By using structural equation modeling, Hollingsworth and Gray [16] found a moderately strong relation between the environment and activity participation for those with mobility impairments. Using qualitative methods, Hammel et al. [15] found that eight major categories of the environment influence participation for individuals with disabilities: built, natural, transportation, assistive technology, information and technology access, social support and societal attitudes, systems and policies, and economic environment. Individuals can experience the same environments differently, however, based on a variety of personal factors, including their phase of post-stroke recovery [13]. Many existing studies focus on individuals within the first three months post-stroke only. Further research is needed to advance our understanding of person-environment interactions that influence specific aspects of participation, such as social participation, for community-dwelling individuals who are further along the post-stroke recovery continuum.

For rehabilitation professionals to adequately address meaningful participation in social activities with their patients after a stroke, there must be a better understanding of how environmental factors affect social participation. The current study addresses this gap by examining how the environment influences social participation for individuals who are at least 6 months post-stroke, including people with aphasia as well as those without aphasia.

## 2. Method

### 2.1. Research Design

This study used a correlational, cross-sectional design [18] to characterize the nature of the relationship between different environmental factors and social participation for individuals post-stroke, as measured by the Activity Card Sort [19].

### 2.2. Participants

Participants were recruited from the Washington University in St. Louis Cognitive Rehabilitation Research Group Stroke Registry, the Aphasia Center at MGH Institute of Health Professions in Boston, and the Stroke Support Group at Spaulding Rehabilitation Hospital in Boston. This research was approved by the Human Research Protection Office at Washington University and the Partners Healthcare Institutional Review Board. Inclusion criteria for all participants were as follows: aged 18 years or older, at least six months post-stroke, able to withstand at least two-three hours of testing by self-report, and mobile enough to travel to the testing site via car or taxicab. Exclusion criteria included history of multiple additional strokes after the initial stroke, traumatic brain injury, history of ongoing seizure disorder, prestroke disability, preexisting neurological condition, or severe medical or psychiatric illness per self-report. Inclusion criteria specifically for individuals with aphasia included acute presence of aphasia by NIH Stroke Scale (NIHSS), aphasia item with a score >0 or current aphasia based on a formal language evaluation conducted within the previous six months, and capability for reliable yes/no responding with communication supports. The total number of participants included in this study were 48 individuals with a complete set of measures, 22 people with aphasia, and 26 people without aphasia. Demographic information of the participants is presented in Table 1.

### 2.3. Setting

The study was conducted onsite at the MGH Institute of Health Professions (MGH IHP) in Boston, Massachusetts and at Washington University School of Medicine in St. Louis, Missouri.

### 2.4. Instruments

Participants were administered an extensive battery of assessments measuring various constructs, including participation, health consequences of stroke, communication skills, cognition, emotional health, and environment, as part of a larger investigation. The current study comprised a selection of these assessments.

#### 2.4.1. Participation Outcome Measure

Participation was measured by the Activity Card Sort [19], a tool that evaluates the extent to which individuals report engaging in a variety of activities in their everyday lives. The ACS contains photographs of 89 activities: 20 instrumental activities of daily living, 35 low-demand leisure tasks, 17 high-demand leisure tasks, and 17 social activities. The ACS is a valid and reliable measure with high internal consistency and sensitivity to detect differences between groups of individuals [19, 20] and has been shown to be adaptable for individuals with aphasia [21]. For this study, percentage retained of prestroke activities in the social realm was the measure of interest, with higher scores indicating that a person has retained more of his or her prestroke activities.

To account for the complexity of the construct of social participation, the outcome was measured in two ways using the ACS: (1) the ACS [19] Social Domain percent retained score, using the assessment's definition of social participation (17 items) that included participating with others and in activities that contribute to society; and (2) the calculated percent retained for items that were ranked high (2 or greater, on a scale of 0 = “none” to 3 = “maximal amount”) on the task demand of needing “a partner to do an activity with” by an independent sample of 43 individuals from a prior unpublished study, which will be referred to as “ACS Partner to Do With” percent retained (11 items), using a narrower definition of social participation that only includes activities that require interaction with another person. This approach allowed us to account for activities that either may have not been included in the Social Domain of the Activity Card Sort, but still were social in nature as indicated by a high ranking in the task demand of “Partner to Do With” or items from the ACS Social Domain that may not have required another to do it with but were considered a social activity. For instance, playing team sports and yard games were two activities ranked high on “Partner to Do With” and, by definition, are social activities as they require interaction with another person, yet are classified by the ACS as high-demand leisure activities. Using ACS Partner to Do With as another outcome measure allowed for a fuller picture of an individual's social participation to be gathered from the ACS than if just items from the Social Domain were examined.

#### 2.4.2. Measures of Environment

The Measure of Stroke Environment (MOSE) [22] is a self-report questionnaire that examines perceived environmental barriers to participation for individuals post-stroke with 47 items in 3 domains: receptivity, built environment, and communication environment. “Receptivity” refers to how receptive other people and institutions in the immediate environment are to others with disability; “built” refers to how the constructed environment poses physical barriers or supports to people with disabilities; and “communication” refers to how the environment provides barriers or supports specifically for people with communication disabilities, such as aphasia. A person's perception for each item is measured on a 4-point rating scale, including ratings for frequency, quality, quantity, importance, satisfaction, choice, and control. The internal consistency of each scale is greater than 0.83. Possible scores ranged from 0 to 57 for the receptivity domain, 0 to 36 for the built environment domain, and 0 to 48 for the communication domain, with higher scores indicating a more positive perception of that aspect of the environment.

The Medical Outcomes Study (MOS) Social Support measure [23] assesses four domains of social support: emotional/informational, tangible, affectionate, and positive social support. There are 19 total items, each with a scale from 1 (none of the time) to 5 (all of the time), with a total possible score ranging from 19 to 95; a higher score indicates more social support. The Positive Social Support domain, a subset of the total scale, was used as a proxy for the social environment in the current study. Internal consistency has been found to be high for this assessment [23].

The Stroke Impact Scale (SIS) [24] consists of eight separate domains that measure self-perceptions of impairments, disabilities, and handicaps for individuals who have had a stroke. In this study, the mobility domain was utilized as an additional proxy for environmental barriers due to mobility limitations. Score ranges from 10 to 50 with higher scores indicating self-perception of higher mobility skills.

#### 2.4.3. Measures of Health Consequences of Stroke

The NIH Stroke Scale (NIHSS) [25] assesses cognitive, sensory, and motor impairments in individuals who have had a stroke. Score ranges from 0 (no detectable impairment) to 42 (maximal impairment). The NIHSS has been found to have good to excellent reliability and high validity [26]. The NIHSS reported in this study is at the time of testing in our lab.

#### 2.4.4. Measures of Communication

For participants with aphasia, the Boston Diagnostic Aphasia Examination 3rd Edition-Short Form (BDAE-3) [27] was used to characterize the severity of aphasia. Three measures of performance were obtained: Expressive component to indicate the participant's ability to produce language, Auditory Comprehension component to indicate the participant's ability to comprehend language, and the Language Competency Index to indicate the overall aphasia involvement. These indices are expressed on a 0 to 100 scale with higher scores indicating better language performance. The entire BDAE-3 has been reported to have high internal consistency with an average alpha of 0.81 [27].

### 2.5. Data Collection

Participants who met all the inclusion and exclusion criteria were assessed during two sessions totaling approximately five to six hours. Participants were administered a consent form at the onset of the session and given multiple-choice questions about the consent process to ensure they understood the purpose and extent of the study. After providing the informed consent, participants were administered the full battery of assessments.

Several modifications were made to the assessments to include individuals with aphasia. The standardized procedure of the ACS only includes one sort; however, the process was split into three separate sorts to minimize confusion among response options for individuals with aphasia [21]. All paper-and-pencil measures in our assessment battery were given in modified presentation format and administration, but not in wording, to be accessible for participants with aphasia [21]. For consistency, participants without aphasia were also administered the modified versions of those tests.

In addition, a hierarchy of support outlined by Tucker et al. [21] was used as a guideline for administering assessments to individuals with aphasia. For all participants, the examiner read the printed question aloud and verified the selected response by pointing to the number and stating the corresponding meaning. If the participant's response was ambiguous or if no response was given, the question and choices were repeated. If necessary, the second level of support involved the examiner simplifying and restating the question and reviewing the choice scale. If necessary, the third level of support was explaining the entire choice scale and repeating the restated question for the participant. If required, the fourth level of support, maximum cueing, was done with the examiner combining a yes-no question with the scale. If the participant was still unable to give an appropriate response after the fourth level of cueing, the examiner moved on to the next question [21]. Further, regardless of the level of support, the response given by the participant was repeated back in terms of the question and verified as being the response intended.

### 2.6. Data Analysis

Data analysis included three steps. First, an independent sample *t*-test was conducted using the Statistical Packages for the Social Sciences, version 21.0 [28] to determine if there was a significant difference between persons with aphasia (PWA) and persons without aphasia (PWOA) on the primary dependent variables, the ACS Social Domain percent retained and ACS Partner to Do With percent retained. The results of the *t*-test determined whether to include group (aphasia/nonaphasia) as a variable in the regression models.

Second, to determine which variables were to be included in the hierarchical regression analyses, Pearson correlations were calculated using SPSS to determine which environmental variables were correlated with the two outcome measures. Only variables significantly correlated (*p* < .05) with either outcome measure were retained for further analysis. This step was taken because no prior literature was available to provide estimates of the effect sizes of the relation between the proposed predictor variables and the outcome variables. We sought to reduce the number of predictor variables to minimize type II error given our modest sample size. Third, using the significantly correlated items as predictor variables, two hierarchical regression analyses were conducted, one for each of the two outcome measures, to determine how much of the variance in percent retained scores was accounted for by environmental factors.

## 3. Results and Discussion

Results by group for the percent retained on the ACS for the Social Domain and for Partner to Do With items are included in Table 2. Both PWA and PWOA had resumed some of their prestroke social activities, but still experienced a notable reduction (about 25 to 30 percent) in social participation in both measures investigated. The results of an independent sample *t*-test (see Table 2) showed no significant difference between PWA and PWOA on ACS Social Domain percent retained, *t*(46) < 1, *p* = 0.59, or on ACS Partner to Do With percent retained *t*(46) < 1, *p* = .98. Therefore, the between group variable (presence or absence of aphasia) was not included in any further analyses.

Table 3 shows the correlations between potential contributor variables and the two social participation outcome measures from the ACS. The MOS Social Support-Positive Social Interactions scale, SIS mobility, and the MOSE receptivity and built domains were found to be significantly correlated with the ACS Social Domain percent retained. Notably, although the magnitude of the correlations with outcome measures varied slightly, the same predictor measures were found to be significantly correlated with ACS Partner to Do With percent retained (see Table 3). Also of note, the NIHSS score and the MOSE communication environment were not found to be associated with the extent of retention of social activities after a stroke.

The results of the hierarchical regression (see Table 4) examining predictors of ACS Social Domain percent retained showed that 37.2% of variance was explained by the stroke severity and environmental variables: MOS Social Support-Positive Social Interactions, SIS mobility, MOSE receptivity domain, and MOSE built environment domain (see Table 4). However, the only environmental variable that was found to be an *independent* predictor of ACS Social Domain percent retained was the MOS Social Support-Positive Social Interactions (*β*-weight = .451; *p* = .001).

The results of the second hierarchical regression analysis (see Table 5) revealed that 34.8% of variance in ACS Partner to Do With percent retained can be explained by the environmental variables: MOS Social Support-Positive Social Interactions, SIS mobility, and MOSE receptivity and built environment domains. As with the first regression analysis, only the MOS Social Support-Positive Social Interactions variable was found to be an *independent* predictor of percent retained for ACS Partner to Do With percent retained (*β*-weight = .464; *p* = .001).

The current study examined how various stroke severity and environmental factors influenced social participation for individuals at least six months post-stroke. A previous finding in the literature indicated that terrain was the major environmental barrier to general participation in daily life for individuals post-stroke [13]. The results of this study show, however, that for social participation, positive social support has more of an influence than any other stroke severity or environmental factor. Although several environmental factors were found to collectively influence social participation, with 37.2% of the variance in the ACS Social Domain percent retained and 34.8% of the variance in ACS Partner to Do With percent retained accounted for by environmental factors, the MOS Social Support-Positive Social Interactions scale was the only independent predictor for either outcome.

The MOS Social Support-Positive Social Interactions scale measures how much a person has someone else to have a good time with, get together for relaxation, or do something enjoyable with; therefore, the availability of these types of social supports appears to be the best predictor of reengaging in social participation after a stroke. This finding is important for several reasons. First, it indicates that social participation may be distinguished from general daily participation and influenced by the environment in a different way. Additionally, it shows that individuals may be able to engage in their meaningful social activities if they have supportive social relationships, regardless of other environmental barriers they may encounter.

A secondary important finding of the study was that there was no statistically significant difference between individuals with and without aphasia on either outcome measure (i.e., ACS Social Domain percent retained and ACS Partner to Do With percent retained). This finding is interesting because by definition, social activities are those where individuals engage in activities with other people [8]; therefore, the communication barrier created by aphasia was expected to manifest as a significant difference in social participation in those with and without aphasia based on prior literature [10]. In this sample of post-stroke individuals, the presence of aphasia did not differentially have an impact upon the level of social participation even though both groups reported significant restrictions in social participation, with more than 20 percent of prestroke activities given up. These results indicate that factors other than simply the presence of a language impairment are more important to consider when examining social participation after a stroke.

Third, it is noteworthy that NIHSS was not a significant correlate of social participation. This finding indicates that the severity of sensory and motor impairments related to stroke did not have a significant relationship to the ability of this group of individuals to participate in social activities. The NIHSS scores in this group of chronic community-dwelling post-stroke survivors were notably low. It is not surprising then that low-level neurological impairment at the chronic stage of recovery did not predict the rather sizable reduction in activity participation post-stroke. However, despite low levels of neurological impairment as measured by the NIHSS, a significant percentage of prestroke activities were given up after a stroke. Although further research is needed to confirm and extend this finding to those with a greater range of neurological impairment, the current results, if taken at face value, imply that the severity of an individual's stroke, as measured by the NIHSS which is heavily dependent upon motor impairment, may not be predictive of their ability to participate socially, if other environmental factors are adequately addressed.

Fourth, the negligible difference between ACS Social Domain percent retained and ACS Partner to Do With percent retained suggests that the presence of another person may not necessarily constitute social participation for individuals who have experienced a stroke. As previously stated, the American Occupational Therapy Association [8] defines social participation as engagement in activities with other people, whether they be family, friends, peers, or the community. The issue is whether active interaction needs to be a part of the social participation equation [2, 29, 30]. For example, when examining this construct specifically for those with aphasia, Dalemans et al. [10] found that social participation is a theoretical concept that individuals with aphasia do not use; rather, they view it more as engagement, involvement, and having a feeling of belonging with others. Results from this study suggest that social participation may or may not explicitly require a partner to do an activity with; an individual may still consider themselves to be engaging in social participation simply by feeling a sense of belonging to a group of people. This is relevant to the current investigation as the ACS contains items in the social domain that both explicitly require interaction with others, such as attending parties, attending family gatherings, and visiting with friends, and items that represent the broader definition of social participation in that they focus on a person's engagement with the world at large, such as eating at a restaurant, going to a place of worship, and traveling. Our results indicate that individuals may still consider an activity that allows them to feel involved on the community level, such as traveling, to be social participation, regardless of whether that activity requires a partner to do it with. This finding must be interpreted with caution, however, because 7 of the 11 Partner to Do With items overlap with the ACS Social Domain activities, and the negligible differences in results for the two outcome measures may be due to this overlap of items in each scale.

### 3.1. Limitations and Future Directions

There were several limitations of this study indicating the need for future research. First, there was a relatively small sample size. Therefore, the generalizability of the results is somewhat limited, and further research with more participants both with and without aphasia is needed to confirm these results. Moreover, the modest sample size leaves open the possibility of a type II error. Our data analytic plan to only include significant correlates of social participation as predictors in the regression analyses somewhat mitigates this possibility, however. This study now provides the literature with effect size estimates via correlation coefficients and beta-weights that can be used to generate power and sample size estimates for future studies.

Second, as mentioned above, there was a significant overlap between the items from the ACS Social Domain and the Partner to Do With items (though not completely so), so it is not surprising that the regression models included the same predictor variables and accounted for a similar percentage of the total variance in the two outcome measures. The similar results for the two outcome measures is an indicator, however, that Positive Social Interactions are a strong contributor to social participation. A third limitation of this study is the nature of the sample itself. Due to the inclusion and exclusion criteria of the study, individuals with more severe impairments of cognition or communication were unable to participate in the study. Therefore, the current sample is skewed toward those with milder stroke and aphasia, although these individuals are likely to represent those who successfully live in the community after a stroke. Finally, the interpretation of our results is limited in that the design was cross-sectional. All “predictor” variables should strictly be interpreted as correlates of the social participation outcome, rather than causal predictors; a future longitudinal approach would address this issue.

### 3.2. Implications for Rehabilitation

The results of this study contribute several unique findings to the literature on participation for individuals post-stroke and have important implications for rehabilitation practice in general. First, these findings can be used to create evidence-based interventions during rehabilitation that target social participation for individuals recovering from stroke. Knowledge of specific environmental factors that influence social participation, including the built environment, the receptivity of the environment, and the positive social support, can help therapists design appropriate interventions to help individuals reengage in their valued activities and social roles. In particular, therapists can emphasize the importance of social support and positive social interactions to clients and their family and friends during the rehabilitation process. If individuals who have had a stroke and their support systems are explicitly told the importance of positive social support, they may be better able to overcome other environmental barriers and may engage in meaningful social activities after discharge. Rehabilitation professionals may choose to direct their intervention efforts toward the environment and lead stroke support groups that encourage clients to participate as a means to increase their positive social support, rather than focusing solely on individual impairments of motor, language, and cognitive systems. Finally, in a broad sense, the results of this study increase the general knowledge on participation in an area beyond self-care activities, which may enable therapists to provide more holistic treatment for clients after a stroke.

## 4. Conclusion

The evidence from this study regarding the extent to which different environmental factors influence social participation will assist rehabilitation therapists to more effectively address meaningful social participation with their clients after a stroke. Addressing social participation during the rehabilitation process will enable individuals to more successfully reintegrate into their daily lives in the community after a stroke. Further, this work sheds light on the importance of social support as an essential modifier of the relationship between the brain and participation. Ultimately, the findings from the current study will add to the general understanding of participation post-stroke, helping to shift the focus of rehabilitation away from remediation of body functions and structures, and toward participation in meaningful activities [31].

## Figures and Tables

**Table 1:** Demographic characteristics of participants with and without aphasia.

Variable	Participants with aphasia (*N* = 22)	Participants without aphasia (*N* = 26)
*Self-reported race and ethnicity*		
Caucasian	11	9
African American	10	17
Hispanic/latino	1	0
*Gender*		
Female	12	17
Male	10	9
*Mean time post-stroke in months (SD)*	38 (56)	17 (7)
*Education in years (SD)*	15 (2.9)	14 (2.2)
*Median NIH Stroke Scale total (SD)*	2 (2.6)	2 (1.9)
*NIH Stroke Scale range*	0-10	0-6
*NIH Stroke Scale 25th/75th percentiles*	1/4	0/3

**Table 2:** Group means and standard deviations in social participation for Activity Card Sort outcomes.

Variables	PWA mean (SD)	PWOA mean (SD)	*p* value
% retained ACS Social Domain	77.4 (20)	74.0 (23)	.59
% retained ACS Partner to Do With	77.0 (26)	69.9 (25)	.98

PWA = persons with aphasia; PWOA = persons without aphasia.

**Table 3:** Correlations of potential predictors with Activity Card Sort outcomes.

Predictors	% retained ACS Social Domain	% retained ACS Partner to Do With
NIHSS	−.14	−.17
SIS mobility	.40^∗∗^	.37^∗^
MOS Social Support-Positive Social Interactions	.53^∗∗^	.54^∗∗^
MOSE receptivity	.36^∗^	.32^∗^
MOSE built environment	.39^∗∗^	.34^∗^
MOSE communication environment	.24	.26

ACS = Activity Card Sort; NIHSS=NIH Stroke Scale; SIS=Stroke Impact Scale; MOS = Medical Outcomes Study; MOSE = Measure of Stroke Environment; ^∗^*p* < .05 and ^∗∗^*p* < .01.

**Table 4:** Multiple regression results for ACS Social Domain predicted by environmental factors.

Predictors	*β*-*Weight*	*p* value
MOS Social Support-Positive Social Interactions	.451	.001^∗^
SIS mobility	.074	.706
MOSE receptivity	.078	.636
MOSE built environment	.189	.390
*R* ^2^	.372	

**Table 5:** Multiple regression results for ACS Partner to Do With predicted by environmental factors.

Predictors	*β*-*Weight*	*p* value
MOS Social Support-Positive Social Interactions	.464	.001^∗^
SIS mobility	.076	.706
MOSE receptivity	.068	.684
MOSE built environment	.144	.519
*R* ^2^	.348	

## Data Availability

The data included in this manuscript are available upon request to the corresponding author.
